# Molecular Genetic Diagnosis of Spinal Muscular Atrophy: Clinical Utility, Challenges, and Lessons Learned from Illustrative Cases in a Single Center

**DOI:** 10.3390/genes17080971

**Published:** 2026-08-19

**Authors:** Jinli Bai, Qinglin Jiang, Hui Jiao, Yuwei Jin, Hong Wang, Xiushan Ge, Ying Gao, Xiaoyin Peng, Fang Song, Yujin Qu, Mei Diao

**Affiliations:** 1 Capital Institute of Pediatrics-Peking University Teaching Hospital, Beijing 100020, China; sunshine046000@163.com; 2 Department of Medical Genetics, Capital Center for Children’s Health, Capital Medical University, Capital Institute of Pediatrics, Beijing 100020, China; 3 Department of Neurology, Capital Center for Children’s Health, Capital Medical University, Capital Institute of Pediatrics, Beijing 100020, China; 4 Department of Dermatology, Capital Center for Children’s Health, Capital Medical University, Capital Institute of Pediatrics, Beijing 100020, China; 5 Department of Pediatric Surgery, Capital Center for Children’s Health, Capital Medical University, Capital Institute of Pediatrics, Beijing 100020, China

**Keywords:** spinal muscular atrophy, genetic testing, compound heterozygous variants, multiplex ligation-dependent probe amplification (MLPA), long-range PCR, long-read sequencing (LRS), ultra-long-read sequencing (Ultra-LRS), allele-specific long-range PCR (AS-LR-PCR)

## Abstract

**Background:** Spinal muscular atrophy (SMA) is mainly caused by biallelic *SMN1* inactivation. While most patients carry homozygous deletions, 3–5% are compound heterozygotes, making molecular diagnosis challenging. **Methods:** A tiered diagnostic strategy was applied to 17 pediatric patients, combining copy number analyses (MLPA and targeted long-read sequencing, tLRS), sequence variant detection (RT-PCR cloning and sequencing, allele-specific long-range PCR with nested PCR, and tLRS), and structural variant analysis (ultra-long-read sequencing, Ultra-LRS). **Results:** Copy numbers were concordant between MLPA and tLRS. MLPA-suggested gene conversions were confirmed by tLRS, while discordant total copy numbers were resolved as large deletions by Ultra-LRS. RT-PCR cloning, and sequencing identified *SMN1* variants in 11/12 cases and confirmed aberrant splicing in three cases, but failed for large deletions. AS-LR-PCR with nested PCR characterized the variants in 13/15 but failed in gene conversion cases. tLRS achieved definitive diagnosis in all cases, and Ultra-LRS precisely delineated breakpoint junctions of two large deletions. **Conclusions:** A hierarchical complementary strategy integrating copy number, sequence, and structural analyses is essential for the accurate diagnosis of compound heterozygous SMA.

## 1. Introduction

Spinal muscular atrophy (SMA) is an autosomal recessive neuromuscular disorder characterized by the progressive degeneration of spinal cord motor neurons, leading to muscle weakness and atrophy [1]. It stands as the leading genetic cause of infant mortality, with an estimated incidence of 1 in 6000~10,000 live births and a carrier frequency of approximately 1 in 40–50 in the general population [2,3,4]. However, recent studies have revealed population-specific variation—e.g., a Romanian cohort reported a carrier frequency of ~1:30, with *SMN1* exon 7 + 8 deletion as the most common pathogenic variant [5], and couple-based screening showed *SMN1* variants were shared by 13.95% of at-risk couples [6]. These findings highlight the need for comprehensive approaches that go beyond simple copy number (CN) detection to identify compound heterozygous *SMN1* variants. The disease exhibits wide clinical variability, primarily classified into four types based on age of onset and disease severity: type I (infantile onset, <6 months), type II (intermediate, 6 to 18 months), type III (juvenile, >18 months), and type IV (adult onset) [7]. With the approval of various drugs, SMA has entered an era of early diagnosis and treatment [8,9].

SMA is caused by the biallelic inactivation of the *SMN1* gene (Survival of Motor Neuron 1, MIM*600354). *SMN2* (Survival of Motor Neuron 2, OMIM*601627), a nearly identical copy of *SMN1*, is a modifier gene of SMA disease severity. Since *SMN1* and *SMN2* reside in a highly complex genomic region on chromosomal 5q13 that is frequently subjected to unequal rearrangements leading to variable CNs of the two genes [1,10,11]. In addition, the two genes share 99.9% sequence homology. And Alu elements and other transposable elements make up over 65% of the human *SMN* gene (*SMN1* or *SMN2*), which spans approximately 44 kb and includes a 10 kb promoter region [12,13]. Apart from hybrid *SMN1*-*SMN2* gene, other structural variants have been characterized, such as partial deletions of *SMN* genes [11,13,14,15,16,17]. Thus, the genotype of SMA is complex and variable. At present, approximately 95% of patients carry homozygous deletion of *SMN1* exon 7 (E7) or exon7/8 (E7/8) [18,19]. The remaining 3~5% of cases are compound heterozygotes with single-nucleotide variations (SNVs), indels or partial deletions on one *SMN1* allele and an absence of the entire gene or gene conversion on the other *SMN1* allele [20,21]. Rare cases are caused by double pathogenic variants in-trans in *SMN1* [22].

The current first-line diagnostic strategies primarily focus on determining *SMN* gene copy number and identifying sequence variations. Copy number detection methods, including multiplex ligation-dependent probe amplification (MLPA) and qPCR, are established as the cornerstone of first-line molecular diagnostics in clinical practice due to the overwhelming prevalence of *SMN1* E7 or E7/8 homozygous deletions, which cause approximately 95% of SMA cases [14,15,16,20,21,22,23,24]. In cases where MLPA yields inconclusive or negative results, additional testing with sequencing technologies is initiated to ensure diagnostic accuracy. RT-cloning and allele-specific long-range PCR (AS-LR-PCR) combined with nested PCR have been utilized for analyzing subtle variants in the *SMN1* gene [20,25,26,27,28,29]. Third-generation sequencing technologies have been developed to analyze *SMN1* [15,17,22,30,31,32,33,34,35]. For example, *SMN*-targeted long-read sequencing (*SMN*-tLRS) on the PacBio platform enables both copy number assessment of *SMN1* and *SMN2* and detection of sequence-level variants. In contrast, Oxford Nanopore Technology (ONT)-based ultra-long-read sequencing (Ultra-LRS) is primarily used to identify complex structural variation (SV) in *SMN1*.

In this study, we performed genetic analyses on 17 pediatric cases of compound heterozygous SMA using various detection methods, including *SMN* gene copy number analysis and *SMN* sequence variant analysis. We aimed to summarize the advantages and limitations of different genetic testing methods for detecting compound heterozygous SMA cases.

## 2. Methods and Material

### 2.1. Samples Collection and Genetic Detection Strategy

A total of 17 patients with a diagnosis of 5q SMA for molecular genetic testing for variants in *SMN1* were included in this study (Supplemental Table S1). Ultra-long DNA was extracted and purified from the peripheral blood according to a previously described protocol [15]. Genomic DNA used for other methods was extracted using either the standard salting-out method or the QIAamp Blood Mini Kit (Qiagen, Hilden, Germany) [22]. Written informed consent was obtained from the parents, and the study was approved by the Institutional Review Board of Capital Institute of Pediatrics (SHERLLM2024036).

**Figure 1:**
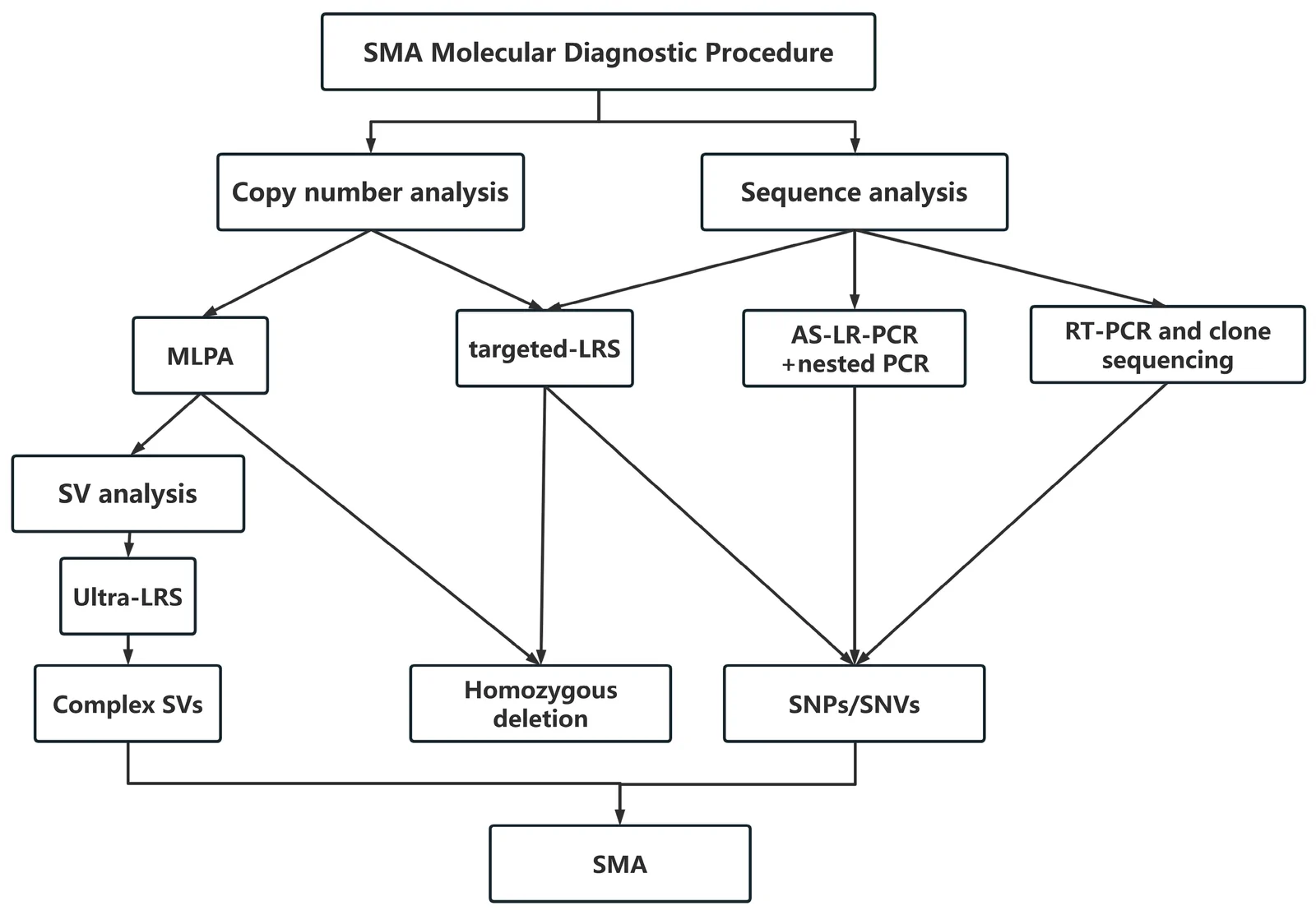
SMA molecular diagnostic procedure.

As illustrated in Figure 1, the molecular diagnostic procedure for SMA includes copy number analysis and sequence analysis, while structural variant analysis is performed for patients with suspected structural variants. Copy number analysis of *SMN1* and *SMN2* was performed using MLPA and tLRS. Sequence analysis encompassed tLRS, AS-LR-PCR combined with nested PCR, and RT-PCR or genomic PCR followed by cloning and Sanger sequencing. When copy number analysis yielded zero copies of *SMN1*, a diagnosis of homozygous deletion SMA was established. In cases where the *SMN1* copy number was one or two, the results were integrated with those from sequence analysis to facilitate the diagnosis of compound heterozygous SMA when SNPs/SNVs were identified. Furthermore, when MLPA results showed discordant copy number calls, Ultra-LRS was employed to resolve complex structural variants (SVs). This integrated approach enabled comprehensive and accurate genetic diagnosis of various SMA genotype.

MLPA, multiplex ligation-dependent probe amplification; AS-LR-PCR, allele-specific long-range PCR; LRS, long-read sequencing; RT-PCR, reverse transcription PCR; SNP/SNV, single-nucleotide polymorphism/variant; SV, structural variant.

### 2.2. Mlpa

The SALSA MLPA Probemix P021 SMA (MRC Holland, Amsterdam, The Netherlands) was used to detect the copy number of *SMN1* and *SMN2* single and total exons in this study according to the manufacturer’s instructions. After denaturation, hybridization, probe connection, and PCR, a 3500xL Dx genetic analyzer (Applied Biosystems, Foster City, CA, USA) was used for detection. The test data were imported into Coffalyser (MRC Holland, Amsterdam, The Netherlands) for further analysis.

The *SMN2-SMN1* conversion gene was first inferred based on the MLPA-P021 assay. A putative conversion gene was considered when the following three criteria were met: (i) the *SMN1* exon 7 (E7) probe signal indicated a single copy of *SMN1*; (ii) the *SMN1* exon 8 (E8) probe signal showed two or more copies; and (iii) the total copy numbers of the *SMN* gene at E7 and E8 were equal (i.e., total E7 copies = total E8 copies). For individuals fulfilling this MLPA pattern, the presence of a *SMN2-SMN1* hybrid gene was further confirmed by tLRS based on Pacbio, characterized by E7 of *SMN2* origin and E8 of *SMN1* origin, corresponding to the *SMN2*^7^/*SMN1*^8^ structure resulting from intergenic gene conversion.

A partial deletion of the *SMN1* gene was suspected when MLPA-P021 revealed discordant copy numbers among different *SMN* exons, such as lower signals in one or more probes relative to other regions. The presence of a partial deletion was subsequently confirmed by tLRS or Gap-PCR.

### 2.3. Smn-Tlrs Based on Pacbio

Genomic DNA samples were subjected to LR-PCR using KOD FX Neo Polymerase (TOYOBO, Japan). Amplification of full-length *SMN1*/*2*, library preparation and SMRT sequencing were performed as previously described [22]. A one-step end-repair and ligation reaction was performed to ligate unique PacBio barcoded adaptors to PCR products, followed by digestion with exonucleases to remove failed ligation DNA. Each reaction mix was purified, quantified and then pooled with equal mass to form the pre-library. Then sequencing primer and polymerase were annealed to the pre-library to obtain the single-molecule real-time dumbbell library using the Sequel Binding Kit 3.2 (Pacific Biosciences, Menlo Park, CA, USA). The sequencing was performed on the Sequel II/IIe platform (Pacific Biosciences, USA) with circular consensus sequencing (CCS) mode. High-fidelity (HiFi) CCS reads were demultiplexed by barcode and aligned to the human reference genome using the SMRT Link software suite (smrtlink 10.1.0.119588, Pacific Biosciences, Menlo Park, CA, USA). *SMN1* and *SMN2* were discriminated by the functional paralogous sequence variant (PSV) at c.840. The aligned CCS reads were employed to analyze *SMN1*/*2* CN, SNVs/indels, and large deletions.

### 2.4. Ultra-LRS Based on Oxford Nanopore Technologies

We performed ONT ultra-LRS to analyze complex SVs involving the *SMN1* gene. DNA extraction, the ultra-long nanopore library and sequencing were used on a Nanopore PromethION (Oxford Nanopore Technologies, Oxford, UK) at the Genome Center of GrandOmics (Wuhan, China) [15]. All reads were aligned to the human reference genome (hg38) to obtain *SMN1* and *SMN2* targets, followed by the PSV at c.840 in E7 to distinguish *SMN1* reads and *SMN2* reads. Structural variation (SV) calls were detected using sniffles [36]. The Integrative Genomics Viewer (IGV) was used to visualize the alignments of wild and mutant molecules.

### 2.5. As-Lr-Pcr Combined with Nested Pcr

The full-length sequence of the *SMN1* and *SMN2* genes was amplified (KOD FX Neo Polymerase, TOYOBO, Osaka, Japan). The LR-PCR method for the specific amplification of *SMN1* was performed using forward primer hybridization-654 bp from the transcription initiation site and a specific *SMN1* E8 reverse primer to amplify a 28.2 kb region that includes exons 1–8 of *SMN1* [25]. Intragenic mutations were detected by sequencing all the *SMN* exons by nested PCR with LR-PCR products as a template. Each *SMN1* exon product was purified with the QIAquick PCR Purfication Kit (Qiagen, Hilden, Germany) and sequenced on an ABI 3130 Genetic Analyzer (Applied Biosystems, Foster City, CA, USA) using the BigDye Terminator v3.1 Cycle Sequencing Kit.

### 2.6. Cloning and Sanger Sequencing

The cloning and sequencing of RT-PCR products was carried out as previously described to detect subtle mutations [26]. Briefly, *SMN* cDNA (exons 1–8) was amplified using primers *SMN*575 and 541C1120, subcloned into the pGEM-T Easy vector (Promega, Madison, WI, USA). *SMN1* and *SMN2* clones were distinguished by restriction enzyme digestion (DraI and DdeI). Approximately 5–8 *SMN1* and 2–3 *SMN2* clones per case were sequenced.

## 3. Result

### 3.1. Copy Number Analysis

**Figure 2:**
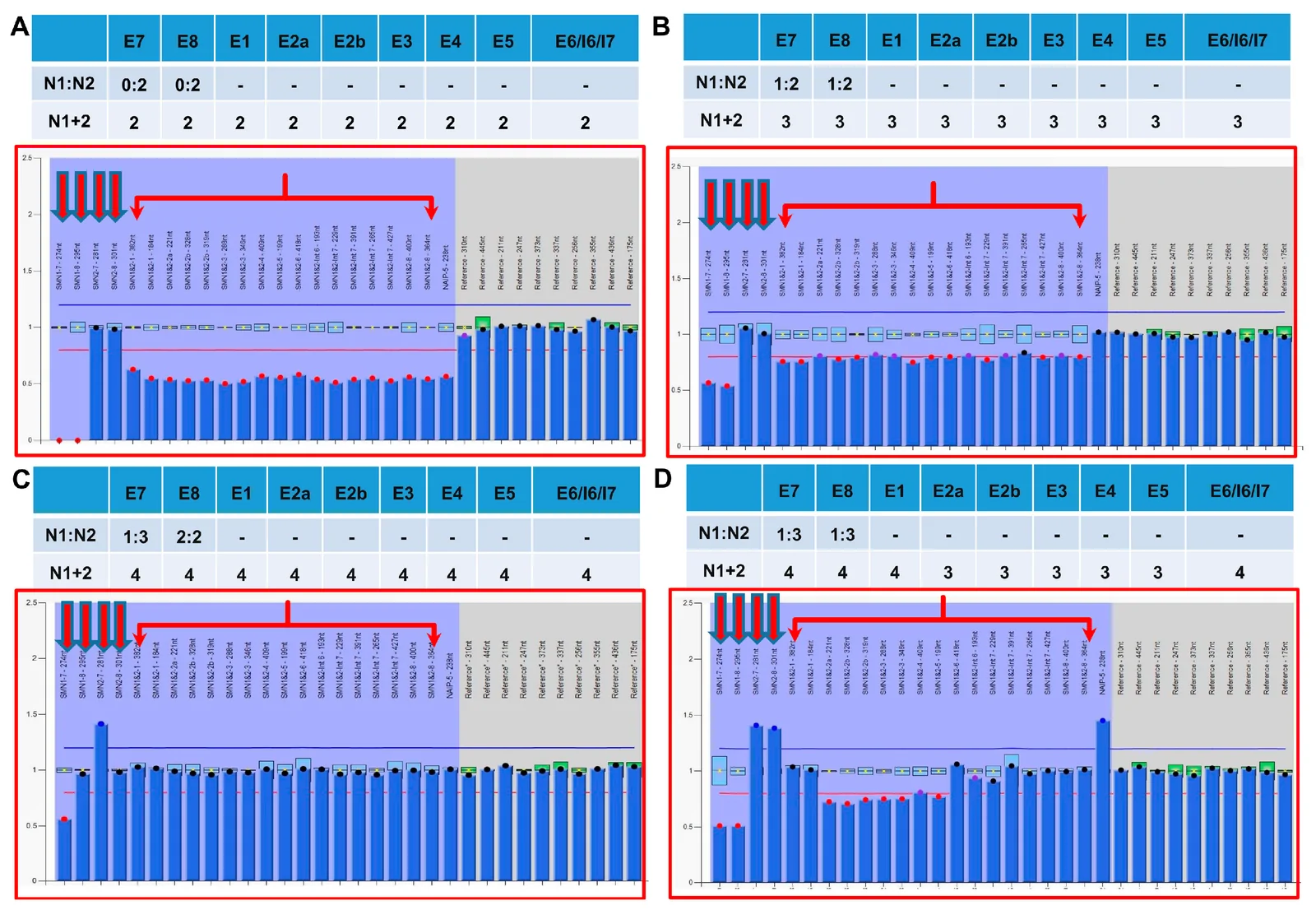
Representative MLPA profiles of *SMN1* and *SMN2* copy number patterns in SMA patients. (**A**) common genotype of homozygous E7 and E8 deletion of *SMN1*, (**B**) concordant total copy numbers across all probes of *SMN1* + *2* in a compound heterozygous SMA patient, (**C**) an *SMN1:SMN2* ratio of 1:3 at E7 and 2:2 at E8, with a total copy number of 4 at both exons, suggesting gene conversion. (**D**) discordant total copy numbers among different *SMN* probe regions. Specifically, the total *SMN* copy number was 3 for E2A-E5, lower than the total copy number of 4 for other *SMN* regions (e.g., E7/E8), suggestive of a partial *SMN1* deletion affecting the E2A-E5. Each MLPA bar graph is enclosed within a red box. The specific probes for *SMN1* E7, *SMN1* E8, *SMN2* E7 and *SMN2* E8 are indicated by red arrows, while non-specific *SMN* probes used for total *SMN* (*SMN1* + *2*) copy number determination are indicated by red horizontal brackets.

According to the MLPA P021 results, we initially classified the SMA patients into distinct genotypes (Supplemental Table S1). Unlike the MLPA profile of homozygous deletion of *SMN1* E7 and 8 (Figure 2A), all patients in this study carried a single copy of *SMN1* E7, as shown in the representative MLPA patterns (Figure 2B–D).

MLPA showed concordant copy numbers across all probes in 15 of 17 cases. Among these, six individuals exhibited a total copy number of 3, with a specific copy number pattern of 1:1:2:2 for *SMN1* E7, *SMN1* E8, *SMN2* E7 and *SMN2* E8 (Figure 2B). The remaining nine cases had a total copy number of 4, which could be further categorized into two distinct patterns: seven cases with a 1:1:3:3 pattern, and two cases with a 1:2:3:2 pattern (Figure 2C). In contrast, two cases displayed discordant copy numbers across different probe sets. Specifically, one case showed a lower copy number for E1 compared to other exons. In another case, MLPA analysis revealed discordant copy numbers among different *SMN* regions (Figure 2D). The total *SMN* (*SMN1* + *2*) copy number was 3 for E2A-E5, but 4 for the other regions (E7 and E8), suggesting a partial deletion affecting the E2A-E5 region.

Targeted-LRS was performed following LR-PCR amplification of full-length *SMN1* and *SMN2* genes. *SMN1* and *SMN2* were distinguished by the functional PSV at c.840, and their respective copy numbers were determined through a haplotype-based algorithm using the read counts of distinct haplotypes. tLRS-based copy number analysis revealed that the copy number ratios of *SMN1* to *SMN2* were 1:2 in six cases and 1:3 in eleven cases.

### 3.2. Sequencing Analysis of SMN Gene

In this study, besides the cloning and Sanger sequencing of RT-PCR products, the genome-based methods comprised two strategies based on targeted long-range amplification of the *SMN1* gene: AS-LR-PCR combined with nested PCR and Sanger sequencing, and tLRS on the PacBio platform.

**Figure 3:**
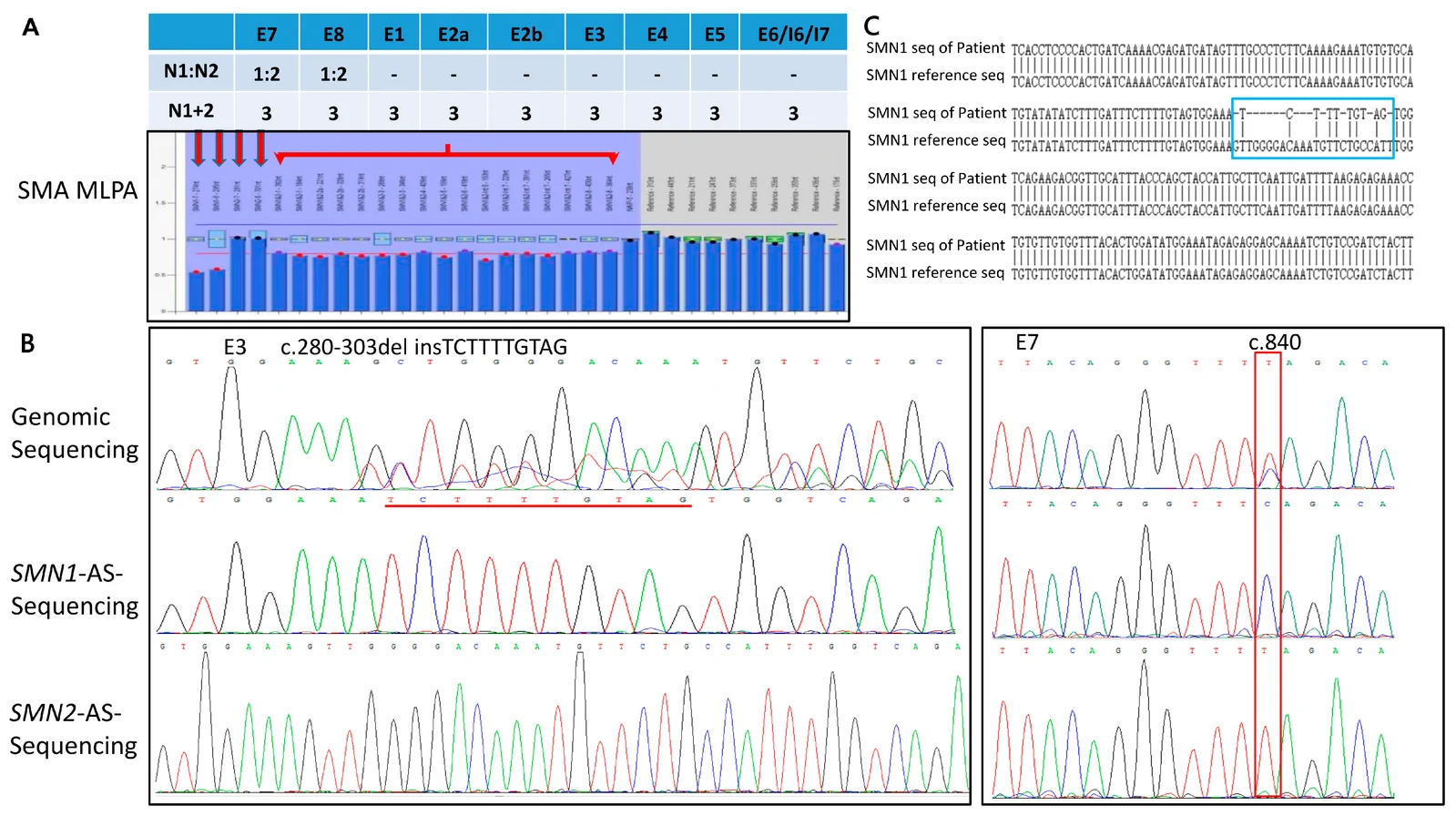
Identification of a novel *SMN1* variant, c.280_303delinsTCTTTTGTAG using combined MLPA and AS-LR-PCR approaches. (**A**), MLPA-P021 analysis shows a single-copy *SMN1* pattern. The specific probes for *SMN1* E7, *SMN1* E8, *SMN2* E7 and *SMN2* E8 are indicated by red arrows, while non-specific *SMN* probes used for total *SMN* (*SMN1* + *SMN2*) copy number determination are indicated by red horizontal brackets. (**B**) Genomic Sanger sequencing reveals double peaks in both E3 and E7. Subsequent *SMN1*-AS-LR-PCR identifies the novel variant, c.280_303delinsTCTTTTGTAG in E3, while the E7 region harbors the *SMN1*-specific base (c.840C). In contrast, *SMN2*-AS-LR-PCR shows no variant in E3, and the E7 region carries the *SMN2*-specific base (c.840T). These findings confirm that the novel variant resides on the single functional SMN1 copy. (**C**), the sequence alignment of *SMN1* further illustrates the position of the variant.

By integrating these sequencing approaches, we achieved a 100% diagnostic yield (17/17) in compound heterozygous SMA cases. In total, 16 distinct *SMN1* variants were identified across the cohort, distributed throughout the entire gene. These variants exhibited diverse mutational types, including three missense variants, five frameshift variants, four nonsense variants, two splicing variants and two large deletions (Table 1). Among these, 15 have been previously documented in the literature or database [20,22,27,37,38,39,40], while one variant, c.280_303delinsTCTTTTGTAG, was novel (Table 1). The novel variant, characterized by an insertion-deletion event involving exon 3, was identified in one case with an *SMN1:SMN2* ratio of 1:2 (Figure 3A). Sanger sequencing revealed double peaks in exons 3 and 7. *SMN1*-AS-LR-PCR identified c.280_303delinsTCTTTTGTAG in exon 3, with the exon 7 region carrying c.840C, whereas *SMN2*-AS-LR-PCR showed no exon 3 variant and carried c.840T at exon 7 (Figure 3B). Sequence alignment further confirmed the variant position (Figure 3C).

Cloning and Sanger sequencing of RT-PCR was performed in the 12 patients with available RNA samples, which revealed the presence of full-length *SMN1* transcripts in eight cases, each carrying variants located in distinct regions. Aberrant truncated *SMN1* transcripts characterized by E7 skipping were identified in three cases (c.835-17_835-14del and c.863G>T). And the large deletion of *SMN1* gene in case 16 was not detected.

**Figure 4:**
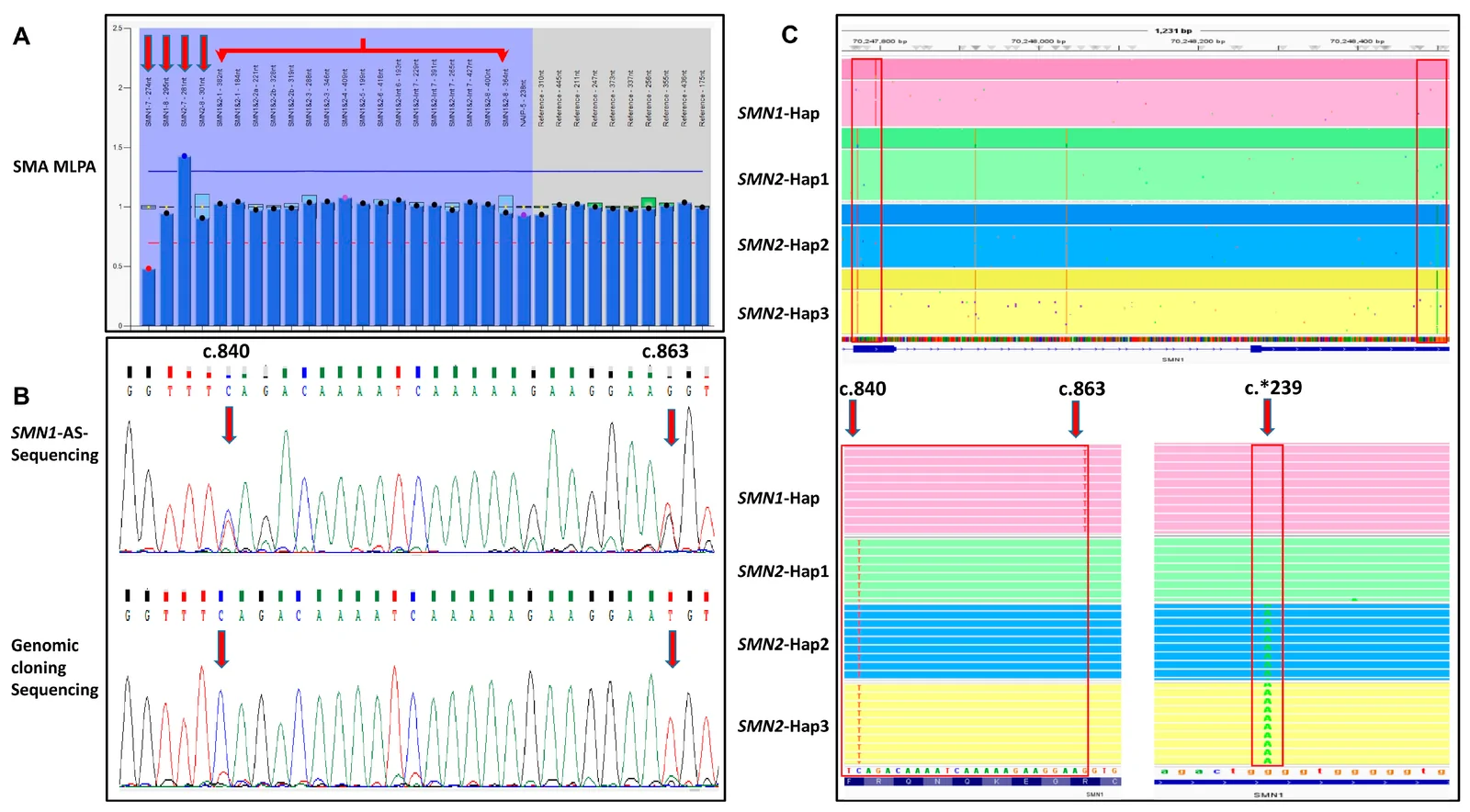
Identification of a *SMN1* c.863G>T variant and a concurrent gene conversion event by integrated sequencing approaches. (**A**) MLPA P021 reveals a copy number pattern of 1:2:3:2 for *SMN1* E7, *SMN1* E8, *SMN2* E7 and *SMN2* E8 (red arrows), suggestive of a gene conversion event involving the E8 PSV (c. *239A>G) in this patient. (**B**) *SMN1*-AS-LR-PCR combined with nested PCR fails to clearly determine the allelic origin of the c.840 (the PSV site) and c.863 (the variant site) loci (red arrows), which is subsequently confirmed by genomic cloning and Sanger sequencing. (**C**) One *SMN1* haplotype and three distinct *SMN2* haplotypes (*SMN2*-Hap1, Hap2 and Hap3) are all captured by tLRS. The red boxes highlight the allelic distribution of three key loci—c.840 (the PSV site distinguishing *SMN1* from *SMN2*), c.863 (the variant site), and c.*239 (the E8 PSV)—across *SMN1* and the three *SMN2* haplotypes. Integrative Genomics Viewer (IGV) shows that the c.863G>T resides on the *SMN1* allele. In addition, the E8 PSV of one *SMN2* copy is converted from the *SMN2*-specific base (adenine, A) to the *SMN1*-specific base (guanine, G), corresponding to c.*239A>G, thereby confirming the gene conversion event.

Of the 15 cases with available DNA, AS-LR-PCR followed by nested PCR identified *SMN1* variants in 11 cases (73.3%). In two cases (cases 3 and 14) involving gene conversion, the variants could not be definitively characterized by this method (Supplemental Table S1). Taking Case 14 as an example, MLPA revealed a specific copy number pattern of 1:2:3:2 for *SMN1* E7, *SMN1* E8, *SMN2* E7 and *SMN2* E8 (Figure 4A), suggesting a gene conversion event involving the E8 PSV. *SMN1*-AS-LR-PCR combined with nested PCR could not accurately determine whether the c.863G>T variant originated from *SMN1* or *SMN2* (Figure 4B). This ambiguity was eventually resolved by cloning and Sanger sequencing (Figure 4B), and tLRS (Figure 4C). In two additional cases (Cases 16 and 17), AS-LR-PCR also failed to fully characterize large structural rearrangements, as this approach relies on exon-by-exon amplification and cannot resolve deletions that extend beyond individual amplicons.

Targeted-LRS on the PacBio platform can resolve variants within the *SMN1* amplification range and established a definitive diagnosis in 17 cases. Among them, the one case with a deletion breakpoint in the upstream region of *SMN1* that fell outside this range, an extended tLRS assay incorporating additional gap primers was used to further clarify the diagnosis.

### 3.3. Structural Variant Analysis

For two cases (Cases 16 and 17) with discordant copy number results from MLPA, ultra-LRS on the ONT platform was performed to resolve structural variants (Table 1 and Table S1). This approach confirmed large deletions in both cases and precisely delineated the breakpoint junctions that were initially suggested. Other intragenic variants were not analyzed using this technique.

## 4. Discussion

In this study, 16 distinct *SMN1* variants were characterized in 17 Chinese patients with compound heterozygous SMA. These variants exhibited considerable heterogeneity, ranging from small-scale sequence alterations (missense, nonsense, frameshift, and splicing variants) to structural rearrangements such as large deletions, and were widely distributed across the *SMN1* gene, spanning most exonic regions as well as intronic sequences. In China, carrier screening studies have reported SMA carrier frequencies of approximately 1/42 to 1.77% (1/56), with the vast majority of carriers exhibiting single-copy *SMN1* exon 7 deletion [41,42]. However, our cohort comprises compound heterozygous patients—a distinct subgroup in which the second pathogenic allele is typically a subtle intragenic mutation or structural rearrangement. Therefore, a more appropriate comparison focuses on the spectrum of point mutations and structural variants. In large Chinese SMA cohorts, the most common point mutations were p.Ser8Lysfs*23 and p.Leu228*, which were also recurrently identified in our study [20,22,37,38]. Additionally, splicing variants such as c.835-5T>G and c.628-3T>G have been reported [29,37], and structural rearrangements including partial deletions and *SMN1*/*SMN2* hybrid genes, though rare, have been documented in Chinese populations [15,22]. Thus, despite our small sample size, the variant spectrum we observed is highly consistent with the established genetic landscape of compound heterozygous SMA in the Chinese population.

The identified variants exhibited marked heterogeneity and wide distribution, encompassing both subtle sequence alterations and large structural rearrangements. Therefore, a comprehensive characterization required the use of multiple complementary genetic testing methodologies. A range of genetic testing methodologies was employed, including MLPA and tLRS for copy number analysis, RT–cloning and sequencing, AS–LR-PCR combined with nested PCR, tLRS for sequence analysis, and ultra-LRS for structural variant analysis. Using the variants identified through these approaches and drawing on illustrative cases, this study provides an in-depth analysis of the key features of each detection method. Particular emphasis is placed on method-specific considerations and applicability, with the goal of helping to avoid the technical pitfalls.

For copy number analysis of *SMN1* genes, MLPA has long been regarded as the primary method and successfully determined the copy number patterns in all 17 SMA patients (100%). Its limitations in compound heterozygous deletion cases have gradually become widely recognized. Since MLPA relies on the hybridization signals of probes targeting distinct regions of the *SMN* genes (especially exons 7 and 8) for copy number quantification, variants located within probe binding sites may interfere with probe annealing, leading to erroneous copy number interpretation. For instance, our group previously reported a case carrying the *SMN1* c.835-17_835-14del variant, which was located precisely within the binding region of the MLPA-P060 probe, resulting in a misinterpretation of *SMN1* E7 copy number [40]. However, this phenomenon was not observed with the P021 kit. Additionally, higher copy numbers (≥4) of the *SMN2* gene may not be accurately quantified. Since the *SMN2* copy numbers in our patients were 2 or 3, we did not observe this phenomenon in this study.

Nevertheless, as the most commonly used first-tier test for SMA, MLPA can provide valuable clues for complex SMA genotypes. Specifically, when the total copy number of all probes is consistent but the *SMN1* E7:*SMN2* E7 ratio differs from the *SMN1* E8:*SMN2* E8 ratio, gene conversion events between *SMN1* and *SMN2* are suggested. In our study, two patients had a total *SMN* copy number of 4, with specific MLPA probe patterns (*SMN1* E7:*SMN1* E8:*SMN2* E7:*SMN2* E8) of 1:2:3:2. The MLPA pattern indicates that one of the three *SMN2* copies carries an exon 8 sequence converted from *SMN1*. This gene conversion event was subsequently confirmed by tLRS. Furthermore, in MLPA analysis, copy number discrepancies across *SMN* probe regions may reflect either genuine deletions or hybridization artifacts. A contiguous multi-probe loss strongly suggests a true partial deletion, whereas an isolated single-probe reduction warrants consideration of both a true localized deletion and SNP affecting probe binding. Thus, the careful interpretation of regional patterns is critical for accurate genotyping. Our observation of two complex *SMN1* structural variants highlights the need to scrutinize subtle MLPA discrepancies, which can inform the selection of sequencing-based approaches for definitive variant characterization.

Currently, several methods are available for sequence variant analysis of the *SMN* gene, including cloning and sequencing of RT-PCR products, AS-LR-PCR combined with nested PCR, and tLRS. Cloning and Sanger sequencing of RT-PCR products from RNA samples allows for the amplification of full-length *SMN* gene transcripts, enabling the detection of exonic variants, particularly those affecting splicing. In this study, this approach successfully identified exon 7 skipping in three cases carrying known splice-altering variants (c.835-17_835-14del and c.863G>T). This method achieved a diagnostic yield of 11/12 (91.7%) among the cases for which RNA samples were available. Its advantage lies in the ability to detect exonic variants, particularly those affecting *SMN1* splicing. However, this method requires high-quality RNA, involves a relatively long procedure, and primarily interrogates exonic regions and exon–intron boundaries. Consequently, it cannot reliably detect deep intronic variants. For comprehensive evaluation, complementary DNA-based LRS covering the entire genomic locus is preferable. As for AS-LR-PCR combined with nested PCR and targeted LRS, they are based on the amplification of the full-length *SMN* genes. Therefore, they require relatively intact DNA templates and are suitable for detecting variants within the amplified region. *SMN1*-AS-LR-PCR combined with nested PCR selectively amplifies the full-length *SMN1* gene using allele-specific PCR, followed by nested PCR amplification of each exon and sequencing, thereby accurately localizing pathogenic variants in a heterozygous background. Among the 15 patients with sufficient DNA samples, this method successfully characterized the variants in 11 cases (73.3%) in our cohort. The optimized protocol reported by Miller et al. successfully mapped several variants (e.g., c.5C>G, p.Ala2Gly) to the *SMN1* gene, which had previously been difficult to resolve due to insufficient amplification specificity [27]. However, the specificity of this technique relies on the recognition of the differential site in *SMN1* E8 by the downstream primer. Consequently, as observed in our study (Cases 3 and 14), when MLPA results indicate a conversion event at the *SMN* gene E8 locus, this technique may fail to clarify the *SMN1* variant. Moreover, this approach is also limited in its ability to detect structural rearrangements, as it primarily relies on nested PCR amplification and Sanger sequencing, which cannot resolve genomic rearrangements beyond the scope of individual amplicons. Indeed, in our cohort, cases 16 and 17, which carried large partial deletions, could not be fully characterized by this method. Therefore, validation by other sequencing approaches, such as tLRS, is necessary for the comprehensive characterization of *SMN1* variants.

Targeted-LRS based on the PacBio, known as the “Comprehensive Analysis of SMA” (CASMA), could amplify the full-length sequences of both *SMN1* and *SMN2*, enabling integrated analysis of copy number quantification and full-length sequence variant detection in a single workflow. Its advantage lies in the ability to directly phase small-scale variants with the full-length gene sequences, thereby determining the gene of origin (*SMN1* or *SMN2*) and the cis-trans configuration of the variants—a capability that is difficult to achieve with traditional methods [20,28,29,30,31]. In this study, CASMA not only confirmed the copy number in all 17 cases (100%) and resolved the ambiguous MLPA patterns by confirming partial *SMN1* deletions in the two discrepant cases, but also finally identified all *SMN1* variants in 17 cases (100%), successfully distinguishing them between *SMN1* and *SMN2*. Notably, CASMA provided intuitive molecular explanations for the discordant copy numbers of exons 7 and 8 observed in MLPA results, such as by identifying gene conversion events involving E8. Although the assay cannot detect variants beyond the coverage of the amplification primers, by utilizing previously described extended amplification primers [20], CASMA was able to detect certain known deletion variants within the *SMN* gene amplification region in our cohort, including the 1415 bp deletion.

Our approaches to *SMN* sequence analysis, including the cloning and Sanger sequencing of RT-PCR products, AS-LR-PCR followed by nested PCR, and tLRS, all enable the simultaneous interrogation of both *SMN1* and *SMN2*, with tLRS in particular providing full-length coverage of both genes. In our cohort, all pathogenic variants were localized to *SMN1*, with no corresponding changes detected in *SMN2*, nor any of the previously reported *SMN2* variants (e.g., c.346A>G, c.835-44A>G, c.835-549A>G, or c.859G>C). This highlights the methodological necessity of parallel *SMN2* interrogation as an internal quality control to exclude *SMN2*-origin variants and avoid false-positive *SMN1* calls. While *SMN2* variants may modify SMA phenotypes, systematic evaluation of these modifiers requires larger cohorts and is beyond the scope of this study, warranting future investigation.

Additionally, for compound heterozygous deletion cases involving large SVs, ultra-LRS based on ONT demonstrates unparalleled advantages [22,34,35,43]. Unlike targeted amplification methods, ONT sequencing does not require pre-designed primers targeting specific regions. Instead, it enables direct sequencing of genomic DNA, allowing for unbiased detection of complex rearrangements spanning the entire *SMN1* gene, including the promoter region. In this study, ultra-LRS successfully characterized the large structural variants in two cases (2/2, 100%) where MLPA suggested discordant copy number patterns—specifically, lower copy numbers of the E1 probe compared to the middle region of the gene—indicating deletion ranges extending beyond the amplification coverage of targeted sequencing approaches. Thus, for patients in whom MLPA or other targeted methods suggest the presence of a large structural variant affecting the terminal regions of *SMN1*, ultra-LRS can be directly employed to definitively characterize the underlying rearrangement.

There are also limitations to this study. Although our cohort encompasses a diverse spectrum of *SMN1* variants that is highly consistent with the known genetic landscape of compound heterozygous SMA in the Chinese population, the sample size (*n* = 17) remains small due to the inherent rarity of this subtype. Larger, multi-center studies are required to validate the diagnostic performance metrics of the proposed strategy, especially for rare or novel variant types. Nonetheless, the diversity of variants characterized and the successful integration of multiple complementary methodologies provide valuable practical insights for the molecular diagnosis of compound heterozygous SMA.

## 5. Conclusions

In summary, these technologies possess distinct advantages and limitations in the detection of compound heterozygous SMA. MLPA is a common and effective copy number analysis method for SMA testing, with preliminary genotyping results guiding the selection of subsequent sequencing methods. tLRS enables simultaneous copy number quantification and sequence variant detection, but it may fail to detect variants located beyond primer coverage. Ultra-LRS effectively resolves complex structural variants, though it requires high-quality DNA and high costs. AS-LR-PCR combined with nested PCR does not require a specialized sequencing platform but is prone to detection pitfalls. In contrast, the cloning and sequencing of RT-PCR are relatively more complex in workflow but offer advantages in detecting aberrant transcripts caused by splicing variants. Thus, they can work together to establish a hierarchical and complementary precision diagnostic framework, which is essential for accurate diagnosis of compound heterozygous SMA.

## Figures and Tables

**Table 1:** Clinical and genetic information from 17 Chinese SMA patients.

Case No.	Gender	Age (Year/Month)	Clinical Type	cDNA Change	Protein Change	Variant Type	Region	References
1	Male	43 y	III	c.5C>G	p.Ala2Gly	missense	Exon 1	[20,22,27]
2	Female	5 m	I	c.22dup	p.Ser8Lysfs*23	frameshift	Exon 1	[20,22,37,38]
3	Male	1 y 5 m	I	c.41_42delinsC	p.Glu14Alafs*16	frameshift	Exon 1	[22]
4	Female	2 y	II	c.188C>A	p.Ser63*	nonsense	Exon 2b	[22]
5	Female	1 y 4 m	I	c.268C>T	p.Gln90*	nonsense	Exon 2b	[39]
6	Female	9 m	I	c.280_303delinsTCTTTTGTAG	p.Val94Serfs*19	frameshift	Exon 3	Novel
7	Female	20 y	III	c.379T>A	p.Tyr127Asn	missense	Exon 3	[22]
8	Male	6 y 10 m	II	c.569G>A	p.Trp190*	nonsense	Exon 4	[22]
9	Female	2 y	II	c.651_652dup	p.pro218Hisfs*26	frameshift	Exon 5	[22]
10	Male	18 y	III	c.683T>A	p.Leu228*	nonsense	Exon 5	[20,22,37,38]
11	Male	15 y	III	c.826T>C	p.Tyr276His	missense	Exon 6	[22]
12	Female	1 y 3 m	I	c.835-17_835-14del	p.Gly279Glufs*5	splicing	Intron 6	[22,40]
13	Female	9 m	I	c.863G>T	p.Gly279Glufs*5	splicing	Exon 7	[20,22,37]
14	Female	4 y 5 m	II	c.863G>T	p.Gly279Glufs*5	splicing	Exon 7	[20,22,37]
15	Male	2 y 5 m	III	c.884A>T	p.*295Leuext*6	frameshift	Exon 7	[22]
16	Male	3 y 5 m	II	Del(chr5:70,924,798-70,926,212), 1415 bp	Not applicable	large deletion	5′UTR-Intron 1	[15,22]
17	Female	1 y 8 m	I	Del(chr5:70,936,243-70,945,220), 8978 bp	Not applicable	large deletion	Exon2–5	[22]

## Data Availability

The original contributions presented in this study are included in the article/Supplementary Materials. Further inquiries can be directed to the corresponding authors.

## Supplementary Materials

The following supporting information can be downloaded at: https://www.mdpi.com/article/10.3390/genes17080971/s1, Table S1: Tiered diagnostic genetic findings in 17 compound heterozygous SMA patients.
